# Face-to-face bullying in and outside of schools and cyberbullying are associated with suicidality in Kenyan high school students: a public health issue

**DOI:** 10.1186/s12888-024-05739-7

**Published:** 2024-04-12

**Authors:** David M. Ndetei, Victoria Mutiso, Jenelle R. Shanley, Christine Musyimi, Pascalyne Nyamai, Timothy Munyua, Tom L. Osborn, Natalie E. Johnson, Sonja Gilbert, Anne Abio, Afzal Javed, Andre Sourander

**Affiliations:** 1 Africa Mental Health Research and Training Foundation, Nairobi, Kenya; 2 Department of Psychiatry, University of Nairobi, Nairobi, Kenya; 3 World Psychiatric Association Collaborating Centre for Research and Training, Nairobi, Kenya; 4 Pacific University, Hillsboro, USA; 5 Shamiri Institute, Nairobi, Kenya; 6 Division of Clinical Epidemiology, Department of Clinical Research, University Hospital Basel, Basel, Switzerland; 7 University of Basel, Basel, Switzerland; 8 Research Centre for Child Psychiatry, Department of Clinical Medicine, Faculty of Medicine, University of Turku, Turku, Finland; 9 Department of Clinical Medicine, Faculty of Medicine, INVEST Child Psychiatry, INVEST Research Flagship Center, University of Turku, Turku, Finland; 10 World Psychiatric Association, Geneva, Switzerland; 11 Department of Child Psychiatry, Turku University Hospital, Turku, Finland

**Keywords:** Bullying, Cyberbullying, Suicidality, Students, Kenyan

## Abstract

**Background:**

Childhood bullying has been classified as a major public health concern by WHO, with negative effects on the health education and social outcomes of both bullies and victims. There is no current Kenyan data on the prevalence of face-to-face bullying and cyberbullying co-occurring in the same cohort of youth and how they are associated with different aspects of suicidality and socio-demographic characteristics. This study aims to fill these gaps in the Kenyan situation so as to inform current policy and practice.

**Methodology:**

This cross-sectional study involved 2,652 students from ten secondary schools in Kenya, selected from three regions representing different levels of public funded schools and socioeconomic spaces. The outcome variable was derived from the questionnaire which asked students questions related to self-harm, suicide thoughts, plans, and attempts. Predictor variables were based on response on experience of bullying in school, out of school, at home, and cyberbullying. Other variables such as gender, age, family background, and class were also collected from the self-reported questions. Data were analyzed using SPSS version 25, with descriptive summary statistics and chi-square tests used to examine variables, and logistic regression analysis used to determine the associations between suicidality and experience of bullying.

**Results:**

The mean age was 16.13 years. More than half of the participants were male, with the largest proportion living in rural areas. Face-to-face bullying was more prevalent than cyberbullying, with 82% of participants experiencing bullying and 68% experiencing it almost daily in the past six months. Both face-to-face bullying and cyberbullying were associated with suicidal thoughts, plans, and attempts. Predictors of suicidal attempts included being bullied outside of school and being a victim of group bullying, while being bullied every day and being bullied by adult men were predictors of suicidal attempts in cyberbullying.

**Conclusion:**

There is a high prevalence of face-to-face bullying both in and outside schools. There is also a high prevalence of cyberbullying. Both face-to-face and cyberbullying are associated with suicidality in Kenyan high school students.

## Introduction

The World Health Organization (WHO) has classified bullying in childhood as a major public health concern [1] with the negative effects of bullying being poor health, educational or social outcomes felt by both the bullies and the victims [2]. A report by the United Nations Educational Scientific and Cultural Organization (UNESCO) shows that globally almost 1 in 3 children has been bullied on one or more days in the preceding month, while 1 in 13 has been bullied on six or more days in the same period [3]. However, the prevalence of bullying in school going children varies widely across the globe with different regions reporting different figures. This prevalence ranges from 27% in a study in Tanzania [4], 29.2% in a Chinese study [5], 82.2% in a Nigerian study [6], and in two Kenyan studies 81.8% and 87.4% [7, 8]. Cyberbullying which is a relatively new type of bullying is becoming rampant, especially in the wake of the COVID-19 pandemic which allowed many school going children to access digital devices and the internet [2, 9]. However, traditional bullying (direct physical and verbal) was reported to have reduced during the COVID-19 pandemic, implying that the pandemic mitigated the bullying rates due to some of the reforms to reduce the virus spread such as small class sizes and online classes without physical interaction [9].

Body shaming, making fun of someone, name calling and group isolation have been identified as the most common forms of childhood bullying [2, 10, 11]. Gender differences have been observed in bullying with studies reporting girls to be more likely bullied compared to boys while boys are more likely to be bullies than girls [9, 12, 13]. However, the opposite has also been reported where boys are victims of bullying more often than girls [6, 10].

Adolescent bullying has been associated with suicidality [4, 5, 13–16]. For instance, a study conducted on 2,647 Chinese adolescents found association between bullying and suicidal ideation, self-harm and suicide attempts with the prevalence of suicidal ideation to be 23.5%, self-harm 6.2% and suicide attempts 4.2% [5]. Additionally, a study that examined the association between cyberbullying victimization and suicidal ideation among school going adolescents in countries in South and Central America and the Caribbean found higher odds of suicidal ideation among adolescents who had experienced cyberbullying victimization than those who had not experienced cyberbullying victimization [17].

Studies conducted across various African countries have also shown that adolescents who experience bullying victimization are more likely to report suicidal thoughts and behaviors, highlighting the critical link between bullying and suicidality [18, 19]. For instance, a study in Tanzania which estimated the prevalence of bullying and its association with suicidal behavior found that being bullied was independently associated with suicidal ideation and suicide attempt [4].

In Kenya, despite the ban on bullying in schools, school going children are still experiencing bullying as evidenced in various studies [7, 8, 11, 12]. A study aimed to investigate the prevalence and frequency of bullying in Nairobi public secondary schools in Kenya revealed that 63.2% to 81.8% of the students had experienced various types of bullying with significant variations based on demographic factors such as sex, age, and school environment [8]. Additionally, other studies conducted across various regions of Kenya on students to explore the determinants, prevalence, and impacts of bullying consistently indicate that bullying is prevalent in Kenya including verbal and physical forms, with significant effects on victims' academic achievement, psychological well-being, and overall school experience [20–23].

Suicidality consists of suicidal ideas with no specific plans to commit suicide; suicidal plans where the individual has already plans on how to commit suicide whereas suicidal attempts are when those plans have been put into effect in one way or another. Suicidality is prevalent in students in Kenya [24]. This can be attributed to socio-economic challenges, the stigma surrounding mental health, and the compounded effects of climate change that students are not immune to [24–27]. There is however a lack of knowledge on the association between face-to-face bullying and cyberbullying with suicidality among high school going students in Kenya. Thus, the general objective of this study is to fill this knowledge gap in Kenya among high school students.

The specific aims are:

1. To determine the current prevalence of face-to-face bullying
2. To determine the prevalence of cyberbullying
3. To determine the associations between the different types of bullying and different stages of suicidality that is, thoughts, plans and attempts
4. To determine the socio-demographic associations with the different types of bullying

## Methods

### Student setting

This cross-sectional study was carried out in 10 government-funded high schools spread across 3 counties in Kenya. A recent study conducted in Kenya had already established relationships and collaborations with the schools and relevant stakeholders within these 3 counties. High school education system in Kenya is typically divided into four forms, thus, data was collected from Form 1, Form 2, Form 3, and Form 4 students. This system is similar to what some countries refer to as grades or years in high school, but in Kenya, they are called “forms.”

### Study participants

This cross-sectional study recruited 2,652 students from ten randomly selected secondary (high) schools to represent the different forms described above. The age range for the students varied from 13 to 20 years due to various factors such as when students began their primary education and whether they repeated any grades. Additionally, students who experience delays or interruptions in their education may also fall within this age range while pursuing their high school studies. The age of 13 to 20 years is the adolescent stage which is a critical period marked by significant changes both physically, psychologically and socially. Individuals in this age may be vulnerable to the negative effects of bullying due to identity formation, body image, peer relationships and increased exposure to social media and online interactions.

### Procedures

#### Sampling

Sampling was multi-stage, where a broad stratification rationale was applied to first select secondary schools that represented each level of the four levels of government-funded schools. In each of the schools, students were allocated to any of the 24 groups headed by a trained research assistant through a permuted block technique. This block randomization aimed to attain a random order but at the same time ensure balanced groups of 15 students.

The study's inclusion criteria were all youths enrolled in the participating schools in the three counties, while exclusion criteria were applied to students not present at schools when collecting the data for varied reasons and those unwilling to participate or give assent/consent. However, none of the students approached refused to assent/consent.

#### Instruments/Measures

The data collection was based on a paper-and-pencil approach, and students were seated in groups of 15 students and supervised by a trained research assistant (RA) in a classroom situation to ensure that there were no consultations among the students. If the student did not understand the question, the RA read it to them up to 3 times and advised them to answer the way they understood the question, that is, the RA did not influence the responses. The following instruments used in this study have been used elsewhere [28].

- 1. A Researcher designed questionnaire to document demographic data. This was assessed using 3 self-reported questions: (1) ‘‘Gender?’’ (Male/Female/Other); (2) “Age? (in years)”; (3) ‘‘In what form (high school grade) are you?’’.
- 2. Bullying questionnaire: The survey comprised a self-administered questionnaire that was adopted by a consortium of experts for use in this study. It has been used in similar surveys of adolescents in 13 European and Asian countries, and explored how traditional bullying and cyberbullying were linked to mental health in youth [5, 28–31]. The widespread use of the tool in cross-cultural studies has been supported by its recognition of the significant overlap that exists between traditional victimization and cyber victimization, as well as resulting psychiatric symptoms.
- 3. The face-to-face bullying experiences were assessed using 11 questions, with 1 question having (yes/no) response: “I have a sibling(s) (half-siblings or similar)” and 10 questions having 4 responses ('Not at all,’ ‘Less than once a week,’ ‘More than once a week,’ ‘Most days’): “How often have you been bullied in school in the past six months?”; “How often have you been bullied outside of school in the past six months?”; “How often have you been bullying others in school in the past six months?”; “How often have you been bullying others outside of school in the past six months?”; “Who has bullied you (at school, outside of school)? - Girls?”; “Who has bullied you (at school, outside of school)? - Boys?”; “Who has bullied you (at school, outside of school)? - Adults?”; “Who has bullied you (at school, outside of school)? - A group (e.g., a group of friends, a class, etc.)?”; “How often have you been bullied by a sibling at home in the past six months?”; “How often have you been bullying your sibling at home in the past six months?”
- 4. Cyberbullying experiences were assessed using 8 questions, with each question having 4 responses ('Never,' 'Less than once a week,' 'More than once a week,' 'Almost every day'): "During the past six months, how often have you been cyberbullied?"; "During the past six months, how often have you cyberbullied others?"; "By who have you been cyberbullied? - Girls?"; "By who have you been cyberbullied? - Boys?”; “By who have you been cyberbullied? - Adult women?”; “By who have you been cyberbullied? - Adult men?”; “By who have you been cyberbullied? - Person unknown to you?”; “By who have you been cyberbullied? - A group (e.g., a group of friends, a class, etc.)?".
- 5. Measure of suicidality- Again the measures of suicidality were part of the consensus agreed upon by the consortium of experts for this study as already explained. This documented suicidal thoughts, plans and attempts. Five questions were asked: (1) “Have you thought seriously about committing suicide?” (‘No, I have not,’ ‘Yes, once,’ ‘Yes, more than once’). For this analysis, the response options were dichotomized into ‘‘No’’ and ‘‘Yes’’; (2) “Have you tried committing suicide?” (‘No, I have not,’ ‘Yes, once,’ ‘Yes, more than once’). For this analysis, the response options were dichotomized into ‘‘No’’and ‘‘Yes’’; (3) “If yes in question 1 above, did you think of a possible way to commit suicide?” (yes/no); (4) “If yes in question 3 above, how?” (list the methods); (5) “If yes in question 2 above, what methods did you use?”. This tool simply asks for the presence or absence of different aspects of suicidality. The questions were added by the Kenyan site to the questionnaire adopted through the process of consultation with all the PIs in the different countries. The questions were borrowed from one of the Kenyan studies [24].

### Data analysis

Data analysis was performed with SPSS version 25 (Armonk, NY: IBM Corp) for Microsoft Windows®. The internal consistency of the questionnaire:- suicidality (3 items), bullying experiences (11 items) and cyberbullying (8 items) were assessed using Cronbach's alpha coefficient after the data collection had been completed.

Descriptive summary statistics in the form of frequency, percentage, mean and standard deviation were generated to examine the variables. The chi-square test was used to inspect the relationships and association between suicidality, bullying variables, and other demographic variables. Fisher's exact test was used to examine the association or independence between gender and cyberbullying. The potential issue of multicollinearity within the model was assessed using the Variance Inflation Factor (VIF) and found low collinearity. Logistic regression analysis was done to determine the associations between suicidality and experience of bullying. The model demonstrated a satisfactory fit when evaluated by the Hosmer and Lemashow goodness-of-fit statistics and diagnostic tests [29]. The choice of binary logistic statistical tests was well considered as the primary aim was to investigate the associations between different types of bullying and distinct stages of suicidality, namely thoughts, plans, and attempts. While Poisson regression in certain contexts is applicable, careful consideration led to opt for logistic regression in this study due to the binary nature of the outcome variables as it is a well-established method for modeling binary outcomes, and its odds ratios provide a meaningful interpretation for the research questions as expressed in the general and specific aims.

### Ethics

Kenyatta University Ethics Review Committee approved the research protocol (#PKU/2456/E1587). A research license was granted from the National Commission for Science, Technology, and Innovation (NACOSTI) (license number NACOSTI/P/22/17173). Permission was sought from institutional heads. This was done in several stages. After the initial ethical clearance, the County Commissioner and the County Director of Education in the three counties were involved to explain to them the nature of the study and that it would benefit the communities through understanding the extent and nature of face-to-face bullying and cyberbullying and that there were no harmful effects of the study. The county-level administrators approved the study and allowed us to engage school principals (institutional heads) with the same explanation. Apart from being school administrators, school principals also act as the direct liaison between the Parent-Teacher Association (PTA) to safeguard the plight of students and school staff. The principal and the PTA work together towards the mutual benefit of schools in Kenya. Part of this cooperation is acting on behalf of parents, a fiduciary arrangement that is logistically feasible as it is challenging to engage each parent spread across different regions. The principal was thus the last contact person in the hierarchical authorization model to approve the request to engage the students. The principals also provided consent on behalf of all parents. Lastly, the high school students were approached with the same explanation, emphasizing that they could withdraw their consent at any time without losing any benefits. Informed written consent was obtained from the students over 18 years and assent from those under 18 years. None of the students approached refused to assent/consent. After completing the questionnaires that the research assistant had issued to students, the students put them in a conveniently placed box to the side of the group. This approach of allowing the students to place the completed tools (irrespective of whether they were fully or partially filled) in the box instead of the RA collecting them enhanced anonymity, privacy, and confidentiality in this study Regarding suicide as this was an anonymous survey, individual students with suicidality were not identified. In mitigation, all students were advised that in the event of suicidal thoughts, plans or attempts to discuss them with trusted friends, teachers, school counselors, and family members. They were also given the option of a helpline at the Africa Mental Health Research and Training Foundation (AMHRTF).

## Results

### Internal consistency

The Cronbach's alpha coefficient for the Suicidality subscale (which had 3 items) was 0.523. For the Bullying Experiences subscale (11 items), Cronbach's alpha was 0.708. The Cronbach's alpha for Cyber Bullying Experience (8 items) was 0.862.

### Social demographics

Table 1 summarizes the socio-demographics of the participants.

**Table 1:** Socio-demographics of the study subjects (*N* = 2652)

Variable	Category	*n* (%)
Gender	Female	862 (33.2)
	Male	1,728 (66.6)
	Other	6 (0.2)
Age (Years)	Mean (SD)	16.13 (1.38)
	Median (IQR)	16.00 (15.00, 17.00)
	Range	13.00, 23.00
Form (high school grade)	1	869 (33.5)
	2	646 (24.9)
	3	729 (28.1)
	4	352 (13.6)
Location of School	Rural	1,627 (61.3)
	Urban	1,025 (38.7)

A total of 2652 high school students participated in the study. The mean (SD) age of the participants was 16.13 years. More than half of the participants were male with the smallest proportion being form 4’s (the final year in high school) (13.6%) and the largest proportion living in rural areas (61.3%).

### Multicollinearity results

These are summarized in Table 2. All the variables were below 5, the highest being 3.23 suggesting that multicollinearity issues are not present among the predictor variables.

**Table 2:** Test of collinearity of independent variables

Variables	Collinearity assessment results
	VIF
Bullying victim in school	1.064
Bullying victim out of school	1.246
Bullying victim Home (Bullied by siblings)	1.214
Cyberbully Victim	2.219
Bully in school	1.813
Bully Out of school	1.825
Bully at Home (Bullying siblings)	2.268
Cyber Bully	2.073
Being a bully and victim (face-to-face)	3.231
Being a cyber-bully and victim	3.172
Gender	1.105

All VIF results were below 5

### Goodness of fit

These are summarized in Table 3. The test revealed a chi-square value of 11.112 and a *p*-value of 0.085. The model fits the data well as *p* > 0.05. This indicates a good model fit.

**Table 3:** Test of goodness of fit

Contingency Table for Hosmer and Lemeshow Test						
		Suicidality_composite = No		Suicidality_composite = Yes		Total
		Observed	Expected	Observed	Expected	
Step 1	1	73	65.61	55	62.39	128
	2	283	275.167	279	286.833	562
	3	82	95.429	139	125.571	221
	4	159	154.773	223	227.227	382
	5	78	80.649	131	128.351	209
	6	58	71.636	158	144.364	216
	7	64	58.429	150	155.571	214
	8	43	38.309	173	177.691	216
**Hosmer and Lemeshow Test**						
	Step	Chi-square	df	Sig		
	1	11.119	6	0.085		

*P* > 0.05

### Bullying and suicidality

#### Different aspects and severity of face-to-face bullying and their associations with suicidality

These are summarized in Table 4. Only 18% of the participants did not experience face-to-face bullying at all that is, 82% experienced bullying with 68% of the bullying taking place almost daily in the past six months. Only 21.2% of the face-to-face bullying took place outside the school. About 16–17% of those bullied had also bullied others either in school or outside school. Boys bullied more than girls (22.9% versus 13.3% respectively) whether in school or outside school. Adults accounted for 16.2% of the bullying while group bullying accounted for 23.2%. In summary, there were more students bullied than there were students who were bullies. Most of those bullied (70–77%) expressed suicidal thoughts. Only a minority (12–14%) of victims of bullying did not express suicidal plans and only 12–18% did not have suicidal attempts.

**Table 4:** Prevalence of different aspects and severity of face-to-face bullying and the different associations with suicidality

	A. Prevalence		B. Suicidality				C. Associations			D. Predictors		
Experiences of Being Bullied	Category	Overall	Total	Suicide Thought^**†**^	Suicide Plan^**†**^	Suicide Attempt^**†**^	Univariate logistic regression			Multivariate logistic regression		
		(*N* = 2652)	(*N* = 2534)	(*n* = 680)	(*n* = 377)	(*n* = 397)	Suicide Thought	Suicide Plan	Suicide Attempt	Suicide Thought	Suicide Plan	Suicide Attempt
							COR (95% CI)	COR (95% CI)	COR (95% CI)	AOR (95% CI)	AOR (95% CI)	AOR (95% CI)
(i) How often have you been bullied in school in the past six months?	Not at all	441 (18.0%)	438 (18.1%)	128 (29.2%)	60 (13.7%)	80 (18.3%)	Ref	Ref	Ref	Ref	Ref	Ref
	Less than once a week	138 (5.6%)	136 (5.6%)	61 (44.9%)	30 (22.1%)	34 (25%)	1.97 (1.32–2.93)***	1.78 (1.08–2.89)*	1.49 (0.94–2.34)	0.92 (0.53–1.60)	0.84 (0.40–1.68)	0.81 (0.41–1.58)
	More than once a week	201 (8.2%)	198 (8.2%)	55 (27.8%)	28 (14.1%)	31 (15.7%)	0.93 (0.64–1.35)	1.04 (0.63–1.67)	0.83 (0.52–1.30)	0.70 (0.42–1.15)	0.70 (0.35–1.34)	0.76 (0.40–1.41)
	Most days	1667 (68.1%)	1647 (68.1%)	407 (24.7%)	246 (14.9%)	229 (13.9%)	0.79 (0.63–1.01)	1.11 (0.82–1.51)	0.72 (0.55–0.96)*	0.93 (0.69–1.27)	1.36 (0.92–2.05)	1.09 (0.74–1.62)
(ii) How often have you been bullied outside of school in the past six months?	Not at all	1929 (78.8%)	1907 (78.8%)	446 (23.4%)	246 (12.9%)	233 (12.2%)	Ref	Ref	Ref	Ref	Ref	Ref
	Less than once a week	323 (13.2%)	321 (13.3%)	131 (40.8%)	73 (22.7%)	90 (28%)	2.26 (1.76–2.89)***	1.99 (1.48–2.65)***	2.80 (2.11–3.69)***	1.28 (0.89–1.83)	1.36 (0.87–2.08)	1.85 (1.21–2.80)**
	More than once a week	103 (4.2%)	100 (4.1%)	46 (46%)	26 (26%)	32 (32%)	2.79 (1.85–4.19)***	2.37 (1.46–3.73)***	3.38 (2.15–5.22)***	1.19 (0.66–2.12)	1.65 (0.84–3.14)	1.59 (0.81–3.03)
	Most days	92 (3.8%)	92 (3.8%)	30 (32.6%)	20 (21.7%)	21 (22.8%)	1.59 (1.00–2.46)*	1.88 (1.10–3.07)*	2.13 (1.25–3.46)**	0.91 (0.47–1.68)	1.11 (0.53–2.22)	1.05 (0.50–2.11)
(iii) How often have you been bullying others in school in the past six months?	Not at all	2045 (83.7%)	2023 (83.7%)	481 (23.8%)	263 (13%)	255 (12.6%)	Ref	Ref	Ref	Ref	Ref	Ref
	Less than once a week	197 (8.1%)	193 (8.0%)	76 (39.4%)	39 (20.2%)	45 (23.3%)	2.08 (1.53–2.82)***	1.69 (1.15–2.44)**	2.11 (1.46–2.99)***	1.18 (0.76–1.82)	1.18 (0.68–1.99)	1.02 (0.59–1.72)
	More than once a week	123 (5.0%)	123 (5.1%)	63 (51.2%)	40 (32.5%)	47 (38.2%)	3.37 (2.33–4.87)***	3.23 (2.15–4.78)***	4.29 (2.90–6.29)***	1.70 (0.97–2.98)	1.70 (0.90–3.15)	1.81 (0.97–3.33)
	Most days	79 (3.2%)	78 (3.2%)	33 (42.3%)	22 (28.2%)	26 (33.3%)	2.35 (1.47–3.72)***	2.63 (1.55–4.32)***	3.47 (2.10–5.60)***	1.18 (0.60–2.25)	1.32 (0.61–2.73)	1.50 (0.72–3.04)
(iv) How often have you been bullying others outside of school in the past six months?	Not at all	2047 (84.0%)	2024 (84.0%)	487 (24.1%)	270 (13.3%)	263 (13%)	Ref	Ref	Ref	Ref	Ref	Ref
	Less than once a week	197 (8.1%)	196 (8.1%)	69 (35.2%)	28 (14.3%)	35 (17.9%)	1.71 (1.25–2.33)***	1.08 (0.70–1.62)	1.46 (0.97–2.12)	1.10 (0.71–1.69)	0.84 (0.48–1.42)	0.75 (0.42–1.30)
	More than once a week	82 (3.4%)	81 (3.3%)	36 (44.4%)	22 (27.2%)	27 (33.3%)	2.52 (1.60–3.95)***	2.42 (1.43–3.96)***	3.35 (2.05–5.36)***	1.20 (0.65–2.20)	1.14 (0.55–2.27)	1.70 (0.86–3.29)
	Most days	111 (4.6%)	109 (4.5%)	53 (48.6%)	38 (34.9%)	47 (43.1%)	2.99 (2.02–4.41)***	3.48 (2.28–5.23)***	5.08 (3.39–7.56)***	1.72 (0.95–3.10)	1.90 (0.98–3.61)	2.49 (1.31–4.69)**
(v) Who has bullied you (at school, outside of school)? – Girls	Not at all	1960 (86.7%)	1935 (86.8%)	475 (24.5%)	266 (13.7%)	259 (13.4%)	Ref	Ref	Ref	Ref	Ref	Ref
	Less than once a week	130 (5.8%)	127 (5.7%)	47 (37%)	21 (16.5%)	32 (25.2%)	1.81 (1.23–2.62)**	1.24 (0.75–1.98)	2.18 (1.41–3.29)***	0.66 (0.38–1.11)	0.43 (0.20–0.83)*	0.70 (0.37–1.28)
	More than once a week	45 (2.0%)	45 (2.0%)	24 (53.3%)	12 (26.7%)	11 (24.4%)	3.51 (1.94–6.42)***	2.28 (1.12–4.36)*	2.09 (1.00–4.05)*	1.53 (0.71–3.30)	0.78 (0.31–1.82)	0.58 (0.22–1.40)
	Most days	125 (5.5%)	123 (5.5%)	55 (44.7%)	35 (28.5%)	42 (34.1%)	2.49 (1.71–3.60)***	2.50 (1.63–3.74)***	3.36 (2.24–4.95)***	1.07 (0.62–1.83)	1.08 (0.58–1.95)	1.09 (0.59–1.97)
(vi) Who has bullied you (at school, outside of school)? – Boys	Not at all	1762 (77.1%)	1740 (77.1%)	397 (22.8%)	219 (12.6%)	217 (12.5%)	Ref	Ref	Ref	Ref	Ref	Ref
	Less than once a week	271 (11.9%)	270 (12.0%)	109 (40.4%)	54 (20%)	64 (23.7%)	2.29 (1.75–2.99)***	1.74 (1.24–2.40)**	2.18 (1.58–2.97)***	1.21 (0.82–1.79)	0.90 (0.55–1.45)	0.85 (0.52–1.37)
	More than once a week	101 (4.4%)	99 (4.4%)	52 (52.5%)	33 (33.3%)	32 (32.3%)	3.74 (2.48–5.65)***	3.47 (2.21–5.36)***	3.35 (2.13–5.19)***	2.36 (1.33–4.20)**	2.04 (1.07–3.83)*	1.66 (0.86–3.13)
	Most days	150 (6.6%)	148 (6.6%)	55 (37.2%)	34 (23%)	39 (26.4%)	2.00 (1.40–2.83)***	2.07 (1.36–3.08)***	2.51 (1.68–3.69)***	1.22 (0.73–2.03)	1.12 (0.61–2.00)	1.10 (0.60–1.98)
(vii) Who has bullied you (at school, outside of school)? – Adults	Not at all	1873 (83.8%)	1847 (83.8%)	438 (23.7%)	241 (13%)	232 (12.6%)	Ref	Ref	Ref	Ref	Ref	Ref
	Less than once a week	154 (6.9%)	153 (6.9%)	61 (39.9%)	33 (21.6%)	37 (24.2%)	2.13 (1.51–2.99)***	1.83 (1.20–2.73)**	2.22 (1.48–3.27)***	1.33 (0.83–2.12)	1.39 (0.80–2.37)	1.29 (0.74–2.19)
	More than once a week	92 (4.1%)	90 (4.1%)	48 (53.3%)	31 (34.4%)	43 (47.8%)	3.68 (2.40–5.66)***	3.50 (2.20–5.48)***	6.37 (4.11–9.85)***	1.41 (0.75–2.63)	1.46 (0.72–2.91)	2.95 (1.52–5.73)**
	Most days	116 (5.2%)	114 (5.2%)	42 (36.8%)	24 (21.1%)	27 (23.7%)	1.88 (1.25–2.77)**	1.78 (1.09–2.80)*	2.16 (1.35–3.35)***	1.05 (0.59–1.82)	1.16 (0.59–2.16)	1.16 (0.60–2.17)
(viii) Who has bullied you (at school, outside of school)?—A group (e.g. a group of friends, a class etc.)	Not at all	1623 (76.8%)	1604 (76.8%)	368 (22.9%)	196 (12.2%)	195 (12.2%)	Ref	Ref	Ref	Ref	Ref	Ref
	Less than once a week	212 (10.0%)	208 (10.0%)	89 (42.8%)	53 (25.5%)	54 (26%)	2.51 (1.86–3.38)***	2.46 (1.73–3.45)***	2.53 (1.78–3.56)***	1.38 (0.92–2.08)	1.93 (1.19–3.09)**	1.90 (1.17–3.05)**
	More than once a week	115 (5.4%)	115 (5.5%)	53 (46.1%)	31 (27%)	35 (30.4%)	2.87 (1.95–4.22)***	2.65 (1.69–4.07)***	3.16 (2.05–4.80)***	1.21 (0.69–2.11)	1.92 (1.01–3.59)*	1.90 (1.00–3.55)*
	Most days	164 (7.8%)	162 (7.8%)	75 (46.3%)	47 (29%)	55 (34%)	2.90 (2.08–4.03)***	2.94 (2.01–4.23)***	3.71 (2.58–5.29)***	1.17 (0.72–1.87)	1.35 (0.77–2.32)	1.68 (0.97–2.85)
(ix) I have sibling(s) (half-siblings or similar)	No	969 (44.0%)	956 (43.9%)	213 (22.3%)	114 (11.9%)	134 (14%)	Ref	Ref	Ref	Ref	Ref	Ref
	Yes	1233 (56.0%)	1220 (56.1%)	380 (31.1%)	215 (17.6%)	204 (16.7%)	1.58 (1.30–1.92)***	1.58 (1.24–2.02)***	1.23 (0.97–1.56)	1.41 (1.11–1.78)**	1.33 (1.00–1.79)	0.96 (0.72–1.29)
(x) How often have you been bullied by a sibling at home in the past six months	Not at all	2132 (88.0%)	2109 (87.9%)	511 (24.2%)	285 (13.5%)	295 (14%)	Ref	Ref	Ref	Ref	Ref	Ref
	Less than once a week	166 (6.9%)	165 (6.9%)	74 (44.8%)	35 (21.2%)	43 (26.1%)	2.54 (1.84–3.51)***	1.72 (1.15–2.53)**	2.17 (1.49–3.11)***	2.15 (1.40–3.29)***	1.41 (0.82–2.35)	1.85 (1.11–3.04)*
	More than once a week	60 (2.5%)	59 (2.5%)	28 (47.5%)	14 (23.7%)	11 (18.6%)	2.82 (1.67–4.76)***	1.99 (1.04–3.58)*	1.41 (0.69–2.64)	2.08 (1.03–4.18)*	1.05 (0.42–2.42)	0.68 (0.23–1.71)
	Most days	66 (2.7%)	65 (2.7%)	32 (49.2%)	23 (35.4%)	20 (30.8%)	3.03 (1.84–4.99)***	3.50 (2.05–5.86)***	2.73 (1.56–4.63)***	1.85 (0.92–3.69)	2.07 (0.96–4.34)	1.74 (0.77–3.79)
(xi) How often have you been bullying your sibling at home in the past six months?	Not at all	1927 (79.6%)	1904 (79.5%)	434 (22.8%)	252 (13.2%)	255 (13.4%)	Ref	Ref	Ref	Ref	Ref	Ref
	Less than once a week	275 (11.4%)	274 (11.4%)	112 (40.9%)	49 (17.9%)	62 (22.6%)	2.34 (1.80–3.04)***	1.43 (1.01–1.98)*	1.89 (1.38–2.57)***	1.19 (0.82–1.71)	0.92 (0.57–1.45)	1.01 (0.63–1.58)
	More than once a week	118 (4.9%)	117 (4.9%)	59 (50.4%)	33 (28.2%)	36 (30.8%)	3.45 (2.36–5.03)***	2.58 (1.67–3.90)***	2.87 (1.88–4.32)***	0.93 (0.53–1.62)	0.83 (0.42–1.58)	0.87 (0.44–1.65)
	Most days	102 (4.2%)	101 (4.2%)	40 (39.6%)	25 (24.8%)	20 (19.8%)	2.22 (1.46–3.34)***	2.16 (1.32–3.41)**	1.60 (0.94–2.60)	1.04 (0.58–1.84)	0.95 (0.48–1.81)	0.54 (0.25–1.11)
(xii) Being a bully and also a victim	No	1795 (71.5%)	1771(71.4%)	389 (22.0%)	212 (12%)	216 (12.2%)	Ref	Ref	Ref	Ref	Ref	Ref
	Yes	717 (28.5%)	710 (28.6%)	279 (39.3%)	158 (22.3%)	168 (23.7%)	2.30(1.91–2.80)***	2.11 (1.67–2.643)*	2.231 (1.80–2.80)***	0.91 (0.48–1.70)	1.41 (0.58–3.42)	1.39 (0.61–3.16)

*COR* Crude Odds Ratio, *AOR* Adjusted Odds Ratio *CI* Confidence Interval, *Ref*. Reference category

^*^p < 0.05

^**^p < 0.01

^***^p < 0.001

^†^ = row percentage

In instances where participants were both bullies and victims, most of them (39.3%) indicated to have suicidal thoughts while 23.7% expressed suicidal attempt with only 12% not planning on suicide (column xii of Table 4).

#### Different aspects and severity of sibling bullying and their associations with suicidality

These results are provided in a narrative. About 12.1% of the participants were bullied by their siblings. Of those bullied by their siblings, 49.2% were having suicidal thoughts almost daily in the past 6 months. Of those who were bullied and had suicidal thoughts, 86.5% had suicidal plans and 86% had suicidal attempts. Most bullies (77.2%-86.6%) expressed suicidal thoughts, plans and attempts.

#### Different aspects and severity of cyberbullying and their associations with suicidality

These are summarized in Table 5. Though cyberbullying occurred less often than face-to-face bullying, it had the same association with suicidality as face-to-face bullying. Of those who were cyberbullies and also victims of cyberbullying, 45.3% expressed suicidal thoughts and only 13.8% did not express suicide attempts.

**Table 5:** Suicidality and Cyber bullying

A. Prevalence			B. Suicidality				C. Associations			D. Predictors		
Cyber bullying	Category	Overall	Total	Suicide Thought^**†**^	Suicide Plan^**†**^	Suicide Attempt^**†**^	Univariate logistic regression			Multivariate logistic regression		
		(*N* = 2652)	(*N* = 2534)	(*n* = 680)	(*n* = 377)	(*n* = 397)	Suicide Thought	Suicide Plan	Suicide Attempt	Suicide Thought	Suicide Plan	Suicide Attempt
							COR (95% CI)	COR (95% CI)	COR (95% CI)	AOR (95% CI)	AOR (95% CI)	AOR (95% CI)
(i) During the past six months, how often have you been cyber bullied?	Never	2073 (84.7%)	2042 (84.6%)	474 (23.2%)	253 (12.4%)	265 (13%)	Ref	Ref	Ref	Ref	Ref	Ref
	Less than once a week	223 (9.1%)	222 (9.2%)	95 (42.8%)	59 (26.6%)	54 (24.3%)	2.47 (1.86–3.29)***	2.56 (1.84–3.53)***	2.16 (1.53–2.99)***	1.28 (0.87–1.89)	1.59 (1.02–2.47)*	1.23 (0.77–1.92)
	More than once a week	93 (3.8%)	92 (3.8%)	45 (48.9%)	26 (28.3%)	28 (30.4%)	3.17 (2.07–4.83)***	2.79 (1.71–4.42)***	2.93 (1.82–4.61)***	1.34 (0.77–2.32)	1.39 (0.74–2.56)	1.29 (0.69–2.36)
	Almost every day	58 (2.4%)	57 (2.4%)	31 (54.4%)	20 (35.1%)	24 (42.1%)	3.94 (2.32–6.75)***	3.82 (2.15–6.62)***	4.88 (2.81–8.35)***	1.65 (0.83–3.31)	1.91 (0.90–3.95)	1.85 (0.88–3.80)
(ii) During the past six months, how often have you cyber bullied others?	Never	1993 (82.6%)	1967 (82.7%)	453 (23%)	250 (12.7%)	244 (12.4%)	Ref	Ref	Ref	Ref	Ref	Ref
	Less than once a week	210 (8.71%)	208 (8.74%)	80 (38.5%)	43 (20.7%)	54 (26%)	2.09 (1.55–2.81)***	1.79 (1.24–2.55)**	2.48 (1.75–3.45)***	1.13 (0.76–1.67)	1.20 (0.75–1.88)	1.11 (0.70–1.75)
	More than once a week	103 (4.27%)	102 (4.29%)	52 (51%)	25 (24.5%)	29 (28.4%)	3.48 (2.32–5.20)***	2.23 (1.37–3.52)***	2.81 (1.76–4.36)***	1.58 (0.94–2.64)	1.09 (0.60–1.95)	1.06 (0.58–1.88)
	Almost every day	106 (4.39%)	102 (4.29%)	48 (47.1%)	29 (28.4%)	37 (36.3%)	2.97 (1.98–4.44)***	2.73 (1.72–4.24)***	4.02 (2.61–6.12)***	1.29 (0.74–2.20)	1.00 (0.53–1.85)	1.31 (0.71–2.35)
(iii) By who have you been cyber bullied?—Girls	Never	1986 (85.9%)	1959 (86.0%)	471 (24%)	258 (13.2%)	261 (13.3%)	Ref	Ref	Ref	Ref	Ref	Ref
	Less than once a week	177 (7.66%)	173 (7.59%)	77 (44.5%)	46 (26.6%)	51 (29.5%)	2.53 (1.84–3.48)***	2.39 (1.65–3.41)***	2.72 (1.90–3.85)***	1.12 (0.71–1.75)	1.29 (0.77–2.13)	0.91 (0.54–1.53)
	More than once a week	44 (1.90%)	43 (1.89%)	16 (37.2%)	8 (18.6%)	7 (16.3%)	1.87 (0.98–3.47)*	1.51 (0.64–3.12)	1.27 (0.51–2.70)	0.72 (0.32–1.55)	0.52 (0.17–1.33)	0.41 (0.15–1.01)
	Almost every day	104 (4.50%)	103 (4.52%)	55 (53.4%)	36 (35%)	40 (38.8%)	3.62 (2.43–5.42)***	3.54 (2.29–5.39)***	4.13 (2.70–6.24)***	1.64 (0.92–2.95)	1.63 (0.86–3.06)	1.15 (0.60–2.16)
(iv) By who have you been cyber bullied? – Boys	Never	1880 (81.8%)	1853 (81.8%)	422 (22.8%)	234 (12.6%)	229 (12.4%)	Ref	Ref	Ref	Ref	Ref	Ref
	Less than once a week	215 (9.35%)	213 (9.40%)	96 (45.1%)	52 (24.4%)	63 (29.6%)	2.78 (2.08–3.72)***	2.23 (1.58–3.12)***	2.98 (2.14–4.11)***	1.60 (1.08–2.36)*	1.19 (0.74–1.88)	1.63 (1.03–2.54)*
	More than once a week	107 (4.65%)	105 (4.64%)	48 (45.7%)	32 (30.5%)	33 (31.4%)	2.86 (1.91–4.25)***	3.03 (1.94–4.66)***	3.25 (2.08–4.98)***	1.14 (0.65–1.98)	1.17 (0.62–2.17)	1.37 (0.73–2.53)
	Almost every day	97 (4.22%)	94 (4.15%)	46 (48.9%)	30 (31.9%)	36 (38.3%)	3.25 (2.13–4.94)***	3.24 (2.03–5.07)***	4.40 (2.82–6.79)***	1.11 (0.59–2.08)	1.19 (0.58–2.38)	1.36 (0.68–2.67)
(v) By who have you been cyber bullied?—Adult women	Never	2038 (89.9%)	2009 (89.9%)	495 (24.6%)	277 (13.8%)	269 (13.4%)	Ref	Ref	Ref	Ref	Ref	Ref
	Less than once a week	100 (4.41%)	97 (4.34%)	43 (44.3%)	21 (21.6%)	33 (34%)	2.44 (1.60–3.68)***	1.73 (1.02–2.80)*	3.34 (2.13–5.14)***	1.11 (0.64–1.91)	0.77 (0.39–1.45)	1.54 (0.84–2.76)
	More than once a week	68 (3.00%)	68 (3.04%)	39 (57.4%)	20 (29.4%)	30 (44.1%)	4.11 (2.53–6.77)***	2.61 (1.49–4.39)***	5.11 (3.09–8.37)***	1.53 (0.80–2.94)	1.15 (0.55–2.32)	1.93 (0.98–3.78)
	Almost every day	60 (2.65%)	60 (2.69%)	29 (48.3%)	24 (40%)	24 (40%)	2.86 (1.70–4.80)***	4.17 (2.42–7.06)***	4.31 (2.50–7.30)***	0.82 (0.40–1.66)	1.91 (0.90–3.98)	1.27 (0.59–2.67)
(vi) By who have you been cyber bullied?—Adult men	Never	2033 (89.5%)	2005 (89.5%)	488 (24.3%)	277 (13.8%)	263 (13.1%)	Ref	Ref	Ref	Ref	Ref	Ref
	Less than once a week	114 (5.02%)	113 (5.04%)	54 (47.8%)	30 (26.5%)	44 (38.9%)	2.85 (1.94–4.17)***	2.25 (1.44–3.45)***	4.22 (2.82–6.28)***	1.22 (0.72–2.06)	1.13 (0.62–2.03)	1.67 (0.94–2.91)
	More than once a week	54 (2.38%)	52 (2.32%)	30 (57.7%)	15 (28.8%)	23 (44.2%)	4.24 (2.43–7.50)***	2.53 (1.33–4.58)**	5.25 (2.97–9.20)***	2.31 (1.10–5.00)*	1.17 (0.51–2.56)	2.40 (1.11–5.11)*
	Almost every day	70 (3.08%)	70 (3.12%)	34 (48.6%)	20 (28.6%)	26 (37.1%)	2.94 (1.81–4.75)***	2.50 (1.43–4.19)***	3.91 (2.34–6.42)***	1.40 (0.73–2.70)	0.98 (0.45–2.05)	1.67 (0.83–3.30)
(vii) By who have you been cyber bullied?—Person unknown to you	Never	1898 (82.7%)	1871 (82.6%)	435 (23.2%)	236 (12.6%)	241 (12.9%)	Ref	Ref	Ref	Ref	Ref	Ref
	Less than once a week	199 (8.67%)	196 (8.66%)	76 (38.8%)	43 (21.9%)	45 (23%)	2.09 (1.53–2.83)***	1.95 (1.34–2.78)***	2.02 (1.39–2.87)***	1.23 (0.82–1.83)	1.18 (0.73–1.88)	1.09 (0.67–1.74)
	More than once a week	94 (4.09%)	93 (4.11%)	48 (51.6%)	32 (34.4%)	35 (37.6%)	3.52 (2.31–5.37)***	3.63 (2.30–5.65)***	4.08 (2.61–6.31)***	1.32 (0.75–2.29)	1.80 (0.97–3.28)	1.30 (0.70–2.38)
	Almost every day	105 (4.57%)	104 (4.59%)	50 (48.1%)	32 (30.8%)	33 (31.7%)	3.06 (2.05–4.56)***	3.08 (1.96–4.73)***	3.14 (2.01–4.82)***	1.31 (0.78–2.19)	1.71 (0.96–2.99)	1.21 (0.68–2.11)
(viii) By who have you been cyber bullied?—A group (e.g. a group of friends, a class etc.)	Never	1857 (83.2%)	1829 (83.1%)	431 (23.6%)	236 (12.9%)	236 (12.9%)	Ref	Ref	Ref	Ref	Ref	Ref
	Less than once a week	158 (7.08%)	156 (7.09%)	68 (43.6%)	38 (24.4%)	46 (29.5%)	2.51 (1.79–3.50)***	2.17 (1.46–3.18)***	2.82 (1.93–4.06)***	1.28 (0.82–1.99)	1.23 (0.73–2.04)	1.28 (0.76–2.12)
	More than once a week	97 (4.34%)	96 (4.36%)	45 (46.9%)	27 (28.1%)	29 (30.2%)	2.86 (1.88–4.33)***	2.64 (1.63–4.16)***	2.92 (1.83–4.57)***	1.17 (0.67–2.03)	1.24 (0.65–2.30)	1.25 (0.67–2.30)
	Almost every day	121 (5.42%)	120 (5.45%)	60 (50%)	36 (30%)	46 (38.3%)	3.24 (2.23–4.72)***	2.89 (1.89–4.34)***	4.20 (2.82–6.19)***	1.53 (0.90–2.59)	1.25 (0.69–2.25)	1.75 (0.98–3.08)
(ix) Being a cyber bully and also a victim	No	2193 (91.4%)	2163 (91.4%)	534(24.7%)	292(13.5%)	299 (13.8%)	Ref	Ref	Ref	Ref	Ref	Ref
	Yes	206 (8.6%)	203 (8.6%)	92 (45.3%)	52 (25.6%)	60(29.6%)	2.528 (1.90–3.40)***	2.20 (1.60–3.1)***	2.62 (1.90–3.60)***	0.427(0.25–0.72)	0.444 (0.24–0.84)	0.43 (0.24–0.80)

*COR* Crude Odds Ratio, *AOR* Adjusted Odds Ratio, *CI* Confidence Interval, *Ref*. Reference category

^*^p < 0.05

^**^p < 0.01

^***^p < 0.001

^†^ = row percentage

#### Different combinations of suicidality in face-to-face bullying

For those that were able to track different combinations of suicidality, most suicidality included the current and past occurrence of thoughts, plans and attempts. However, a smaller number of those who attempted suicide had not expressed thoughts but made plans before the attempt. A smaller number of those who attempted suicide did not express suicidality thoughts or make plans. The trends were similar for both face-to-face bullying. There were significant (p < 0.05) associations with various types of bullying and various combinations of thoughts, plans and attempts for face-to-face bullying and in particular where bullying occurred almost every day. See Table 6 for details of these associations.

**Table 6:** Suicidality progression in face-to-face Bullying

A. Experience		B. Pathways				C. Association			D. Predictors		
Experiences of Being Bullied	Category	Total (*N* = 2,534)	Thoughts + Plans + Attempts^**†**^	Thoughts + No Plans + Attempts^**†**^	No Thoughts + No Plans + Attempts^**†**^	Univariate logistic regression			Multivariate logistic regression		
			(*n* = 253)	(*n* = 96)	(*n* = 48)	Thoughts + Plans + Attempts	Thoughts + No Plans + Attempts	No Thoughts + No Plans + Attempts	Thoughts + Plans + Attempts	Thoughts + No Plans + Attempts	No Thoughts + No Plans + Attempts
						COR (95% CI)	COR (95% CI)	COR (95% CI)	AOR (95% CI)	AOR (95% CI)	AOR (95% CI)
(i) How often have you been bullied in school in the past six months?	Not at all	438 (18.1%)	43 (9.8%)	26 (5.9%)	11 (2.5%)	Ref	Ref	Ref	Ref	Ref	Ref
	Less than once a week	136 (5.62%)	22 (16.2%)	9 (6.6%)	3 (2.2%)	1.77 (1.00–3.06)*	1.12 (0.49–2.37)	0.88 (0.20–2.85)	0.88 (0.37–1.98)	1.23 (0.42–3.34)	0.29 (0.01–2.23)
	More than once a week	198 (8.19%)	20 (10.1%)	8 (4%)	3 (1.5%)	1.03 (0.58–1.78)	0.67 (0.28–1.44)	0.60 (0.13–1.94)	0.83 (0.37–1.77)	0.92 (0.31–2.46)	0.70 (0.09–3.70)
	Most days	1,647 (68.1%)	156 (9.5%)	46 (2.8%)	27 (1.6%)	0.96 (0.68–1.39)	0.46 (0.28–0.75)**	0.65 (0.33–1.37)	1.38 (0.86–2.30)	0.72 (0.38–1.42)	1.23 (0.47–3.62)
(ii) How often have you been bullied outside of school in the past six months?	Not at all	1,907 (78.8%)	155 (8.1%)	55 (2.9%)	23 (1.2%)	Ref	Ref	Ref	Ref	Ref	Ref
	Less than once a week	321 (13.3%)	53 (16.5%)	20 (6.2%)	17 (5.3%)	2.24 (1.58–3.11)***	2.24 (1.29–3.72)**	4.58 (2.38–8.63)***	1.59 (0.96–2.60)	1.11 (0.49–2.41)	4.97 (1.80–13.3)**
	More than once a week	100 (4.13%)	20 (20%)	8 (8%)	4 (4%)	2.83 (1.65–4.65)***	2.93 (1.26–6.00)**	3.41 (0.99–9.09)*	1.66 (0.77–3.44)	1.09 (0.30–3.29)	1.87 (0.22–9.96)
	Most days	92 (3.80%)	16 (17.4%)	4 (4.3%)	1 (1.1%)	2.38 (1.31–4.08)**	1.53 (0.46–3.84)	0.90 (0.05–4.34)	1.39 (0.60–2.99)	0.73 (0.11–2.79)	0.62 (0.03–4.61)
(iii) How often have you been bullying others in school in the past six months?	Not at all	2,023 (83.7%)	162 (8%)	63 (3.1%)	30 (1.5%)	Ref	Ref	Ref	Ref	Ref	Ref
	Less than once a week	193 (7.99%)	28 (14.5%)	11 (5.7%)	6 (3.1%)	1.95 (1.24–2.96)**	1.88 (0.92–3.49)	2.13 (0.79–4.84)	1.33 (0.71–2.42)	0.65 (0.21–1.75)	0.83 (0.16–3.19)
	More than once a week	123 (5.09%)	32 (26%)	9 (7.3%)	6 (4.9%)	4.04 (2.59–6.17)***	2.46 (1.12–4.82)*	3.41 (1.26–7.80)**	1.91 (0.93–3.76)	1.56 (0.53–4.12)	0.58 (0.10–2.65)
	Most days	78 (3.23%)	19 (24.4%)	4 (5.1%)	3 (3.8%)	3.70 (2.10–6.24)***	1.68 (0.50–4.21)	2.66 (0.63–7.68)	1.69 (0.72–3.74)	0.96 (0.19–3.50)	1.73 (0.29–7.97)
(iv) How often have you been bullying others outside of school in the past six months?	Not at all	2,024 (84.0%)	174 (8.6%)	60 (3%)	29 (1.4%)	Ref	Ref	Ref	Ref	Ref	Ref
	Less than once a week	196 (8.13%)	16 (8.2%)	12 (6.1%)	7 (3.6%)	0.95 (0.53–1.57)	2.13 (1.08–3.90)*	2.55 (1.02–5.58)*	0.70 (0.34–1.33)	1.04 (0.37–2.56)	0.98 (0.19–3.71)
	More than once a week	81 (3.36%)	19 (23.5%)	5 (6.2%)	3 (3.7%)	3.26 (1.86–5.47)***	2.15 (0.74–5.03)	2.65 (0.62–7.65)	1.61 (0.74–3.37)	1.22 (0.33–3.84)	2.94 (0.48–14.2)
	Most days	109 (4.52%)	31 (28.4%)	10 (9.2%)	6 (5.5%)	4.23 (2.68–6.53)***	3.31 (1.55–6.38)***	4.01 (1.48–9.24)**	1.87 (0.90–3.82)	1.89 (0.58–5.58)	3.32 (0.76–13.0)
(v) Who has bullied you (at school, outside of school)? – Girls	Not at all	1,935 (86.8%)	166 (8.6%)	65 (3.4%)	28 (1.4%)	Ref	Ref	Ref	Ref	Ref	Ref
	Less than once a week	127 (5.70%)	19 (15%)	10 (7.9%)	3 (2.4%)	1.87 (1.09–3.06)*	2.46 (1.16–4.70)*	1.65 (0.39–4.73)	0.61 (0.28–1.25)	0.76 (0.23–2.08)	1.57 (0.31–5.83)
	More than once a week	45 (2.02%)	9 (20%)	2 (4.4%)	0 (0%)	2.66 (1.19–5.39)*	1.34 (0.22–4.47)	0.00 (0.00–3,480,318)	0.83 (0.29–2.08)	0.35 (0.02–2.01)	0.00 (0.00–392,669,485,947,819,905,046,668,824)
	Most days	123 (5.52%)	25 (20.3%)	10 (8.1%)	7 (5.7%)	2.72 (1.67–4.27)***	2.55 (1.20–4.87)**	4.11 (1.62–9.11)**	0.89 (0.42–1.76)	0.72 (0.18–2.16)	3.48 (0.98–11.0)*
(vi) Who has bullied you (at school, outside of school)? – Boys	Not at all	1,740 (77.1%)	136 (7.8%)	54 (3.1%)	27 (1.6%)	Ref	Ref	Ref	Ref	Ref	Ref
	Less than once a week	270 (12.0%)	39 (14.4%)	17 (6.3%)	8 (3%)	1.99 (1.34–2.89)***	2.10 (1.16–3.60)**	1.94 (0.81–4.12)	0.96 (0.54–1.66)	1.05 (0.42–2.46)	0.43 (0.10–1.56)
	More than once a week	99 (4.39%)	23 (23.2%)	5 (5.1%)	4 (4%)	3.57 (2.13–5.79)***	1.66 (0.57–3.87)	2.67 (0.78–7.00)	1.99 (0.96–4.00)	0.77 (0.18–2.62)	1.01 (0.17–4.58)
	Most days	148 (6.56%)	25 (16.9%)	10 (6.8%)	4 (2.7%)	2.40 (1.48–3.76)***	2.26 (1.07–4.35)*	1.76 (0.52–4.58)	1.04 (0.51–2.05)	0.99 (0.31–2.74)	1.17 (0.24–4.71)
(vii) Who has bullied you (at school, outside of school)? – Adults	Not at all	1,847 (83.8%)	147 (8%)	57 (3.1%)	28 (1.5%)	Ref	Ref	Ref	Ref	Ref	Ref
	Less than once a week	153 (6.94%)	23 (15%)	10 (6.5%)	4 (2.6%)	2.05 (1.25–3.23)**	2.20 (1.04–4.21)*	1.74 (0.51–4.52)	1.45 (0.76–2.66)	1.12 (0.37–2.90)	0.69 (0.13–2.76)
	More than once a week	90 (4.08%)	27 (30%)	11 (12.2%)	5 (5.6%)	4.96 (3.02–7.94)***	4.37 (2.10–8.36)***	3.82 (1.27–9.35)**	1.74 (0.80–3.65)	3.39 (1.18–9.29)*	2.04 (0.40–9.00)
	Most days	114 (5.17%)	20 (17.5%)	5 (4.4%)	2 (1.8%)	2.46 (1.44–4.02)***	1.44 (0.49–3.34)	1.16 (0.19–3.93)	1.52 (0.73–3.02)	0.56 (0.08–2.22)	0.68 (0.09–3.35)
(viii) Who has bullied you (at school, outside of school)?—A group (e.g. a group of friends, a class etc.)	Not at all	1,604 (76.8%)	122 (7.6%)	50 (3.1%)	23 (1.4%)	Ref	Ref	Ref	Ref	Ref	Ref
	Less than once a week	208 (9.96%)	36 (17.3%)	11 (5.3%)	7 (3.4%)	2.54 (1.68–3.77)***	1.74 (0.85–3.27)	2.39 (0.94–5.38)*	2.01 (1.14–3.49)*	1.02 (0.37–2.49)	2.61 (0.76–8.33)
	More than once a week	115 (5.51%)	23 (20%)	9 (7.8%)	3 (2.6%)	3.04 (1.82–4.90)***	2.64 (1.19–5.26)**	1.84 (0.43–5.39)	2.00 (0.96–4.03)	1.63 (0.49–4.76)	0.85 (0.09–5.08)
	Most days	162 (7.75%)	36 (22.2%)	14 (8.6%)	5 (3.1%)	3.47 (2.27–5.21)***	2.94 (1.53–5.31)***	2.19 (0.73–5.40)	1.40 (0.73–2.61)	1.99 (0.75–4.85)	1.15 (0.25–4.24)
(ix) I have sibling(s) (half-siblings or similar)	No	956 (43.9%)	76 (7.9%)	35 (3.7%)	23 (2.4%)	Ref	Ref	Ref	Ref	Ref	Ref
	Yes	1,220 (56.1%)	138 (11.3%)	46 (3.8%)	20 (1.6%)	1.48 (1.10–1.99)**	1.03 (0.66–1.62)	0.68 (0.37–1.24)	1.14 (0.80–1.62)	1.03 (0.61–1.76)	0.39 (0.17–0.88)*
(x) How often have you been bullied by a sibling at home in the past six months	Not at all	2,109 (87.9%)	190 (9%)	73 (3.5%)	32 (1.5%)	Ref	Ref	Ref	Ref	Ref	Ref
	Less than once a week	165 (6.88%)	23 (13.9%)	10 (6.1%)	10 (6.1%)	1.64 (1.00–2.56)*	1.80 (0.86–3.40)	4.19 (1.92–8.38)***	1.27 (0.66–2.32)	2.09 (0.87–4.64)	2.98 (0.90–8.67)
	More than once a week	59 (2.46%)	9 (15.3%)	2 (3.4%)	0 (0%)	1.82 (0.82–3.58)	0.98 (0.16–3.22)	0.00 (0.00–51,114,092,156)	0.70 (0.19–2.01)	1.21 (0.18–4.75)	0.00 (0.00–456,310,851,852,091,581,802,446)
	Most days	65 (2.71%)	17 (26.2%)	2 (3.1%)	1 (1.5%)	3.58 (1.96–6.22)***	0.89 (0.14–2.91)	1.01 (0.06–4.83)	2.06 (0.86–4.66)	0.79 (0.11–3.38)	1.08 (0.05–8.43)
(xi) How often have you been bullying your sibling at home in the past six months?	Not at all	1,904 (79.5%)	168 (8.8%)	57 (3%)	30 (1.6%)	Ref	Ref	Ref	Ref	Ref	Ref
	Less than once a week	274 (11.4%)	33 (12%)	20 (7.3%)	9 (3.3%)	1.41 (0.94–2.08)	2.55 (1.47–4.25)***	2.12 (0.94–4.34)	0.84 (0.47–1.44)	1.22 (0.53–2.60)	1.38 (0.41–4.07)
	More than once a week	117 (4.88%)	25 (21.4%)	8 (6.8%)	3 (2.6%)	2.81 (1.72–4.43)***	2.38 (1.03–4.84)*	1.64 (0.39–4.70)	0.90 (0.41–1.85)	0.84 (0.25–2.43)	0.81 (0.13–4.06)
	Most days	101 (4.22%)	16 (15.8%)	3 (3%)	1 (1%)	1.95 (1.08–3.31)*	0.99 (0.24–2.75)	0.62 (0.03–2.96)	0.56 (0.22–1.24)	0.89 (0.19–2.94)	0.35 (0.02–2.52)

*COR* Crude Odds Ratio, *AOR* Adjusted Odds Ratio, *CI* Confidence Interval

^*^p < 0.05

^**^p < 0.01

^***^p < 0.001

^†^ = row percentage

#### Different combinations of suicidality in cyberbullying

These are summarized in Table 7. The trends are similar to those in face-to-face bullying.

**Table 7:** Suicidality progression in Cyber bullying

A. Experience			B. Pathways			C. Association			D. Predictors		
Cyber bullying	Category	Total (*N* = 2,534)	Thoughts + Plans + Attempts^**†**^	Thoughts + No Plans + Attempts^**†**^	No Thoughts + No Plans + Attempts^**†**^	Univariate logistic regression			Multivariate logistic regression		
			(*n* = 253)	(*n* = 96)	(*n* = 48)	Suicide—Thoughts + Plans + Attempts	Suicide—Thoughts + No Plans + Attempts	Suicide—No Thoughts + No Plans + Attempts	Suicide—Thoughts + Plans + Attempts	Suicide—Thoughts + No Plans + Attempts	Suicide—No Thoughts + No Plans + Attempts
						COR (95% CI)	COR (95% CI)	COR (95% CI)	AOR (95% CI)	AOR (95% CI)	AOR (95% CI)
(i) During the past six months, how often have you been cyber bullied?	Never	2,042 (84.6%)	168 (8.2%)	59 (2.9%)	38 (1.9%)	Ref	Ref	Ref	Ref	Ref	Ref
	Less than once a week	222 (9.20%)	37 (16.7%)	13 (5.9%)	4 (1.8%)	2.23 (1.50–3.25)***	2.09 (1.08–3.75)*	0.97 (0.29–2.44)	1.42 (0.84–2.37)	0.82 (0.33–1.90)	0.95 (0.19–3.40)
	More than once a week	92 (3.81%)	16 (17.4%)	10 (10.9%)	2 (2.2%)	2.35 (1.30–4.01)**	4.10 (1.92–7.97)***	1.17 (0.19–3.91)	1.17 (0.54–2.38)	1.75 (0.63–4.56)	0.78 (0.10–3.76)
	Almost every day	57 (2.36%)	17 (29.8%)	6 (10.5%)	1 (1.8%)	4.74 (2.57–8.41)***	3.95 (1.47–8.92)**	0.94 (0.05–4.47)	2.50 (1.12–5.40)*	1.26 (0.31–4.21)	0.17 (0.01–1.52)
(ii) During the past six months, how often have you cyber bullied others?	Never	1,967 (82.7%)	163 (8.3%)	52 (2.6%)	29 (1.5%)	Ref	Ref	Ref	Ref	Ref	Ref
	Less than once a week	208 (8.74%)	28 (13.5%)	15 (7.2%)	11 (5.3%)	1.72 (1.10–2.61)*	2.86 (1.53–5.06)***	3.73 (1.76–7.38)***	0.93 (0.53–1.59)	1.09 (0.46–2.42)	2.37 (0.69–7.09)
	More than once a week	102 (4.29%)	18 (17.6%)	10 (9.8%)	1 (1%)	2.37 (1.35–3.96)**	4.00 (1.87–7.81)***	0.66 (0.04–3.14)	1.04 (0.52–1.99)	1.46 (0.51–3.82)	0.32 (0.01–2.21)
	Almost every day	102 (4.29%)	23 (22.5%)	10 (9.8%)	4 (3.9%)	3.22 (1.93–5.19)***	4.00 (1.87–7.81)***	2.73 (0.80–7.10)	1.20 (0.59–2.35)	2.00 (0.71–5.23)	0.49 (0.06–2.46)
(iii) By who have you been cyber bullied?—Girls	Never	1,959 (86.0%)	167 (8.5%)	59 (3%)	35 (1.8%)	Ref	Ref	Ref	Ref	Ref	Ref
	Less than once a week	173 (7.59%)	30 (17.3%)	17 (9.8%)	4 (2.3%)	2.25 (1.45–3.40)***	3.51 (1.94–6.04)***	1.30 (0.39–3.30)	0.95 (0.51–1.71)	1.20 (0.47–2.87)	0.40 (0.05–1.89)
	More than once a week	43 (1.89%)	5 (11.6%)	2 (4.7%)	0 (0%)	1.41 (0.48–3.32)	1.57 (0.25–5.28)	0.00 (0.00–1,157,168)	0.52 (0.16–1.44)	0.59 (0.08–2.60)	0.00 (0.00–76,892,936,493)
	Almost every day	103 (4.52%)	25 (24.3%)	10 (9.7%)	5 (4.9%)	3.44 (2.10–5.47)***	3.46 (1.62–6.70)***	2.80 (0.95–6.71)*	1.08 (0.51–2.21)	1.16 (0.36–3.37)	1.06 (0.22–4.80)
(iv) By who have you been cyber bullied?—Boys	Never	1,853 (81.8%)	148 (8%)	49 (2.6%)	32 (1.7%)	Ref	Ref	Ref	Ref	Ref	Ref
	Less than once a week	213 (9.40%)	39 (18.3%)	19 (8.9%)	5 (2.3%)	2.58 (1.74–3.76)***	3.61 (2.03–6.15)***	1.37 (0.46–3.25)	1.43 (0.84–2.41)	2.50 (1.12–5.40)*	0.54 (0.08–2.36)
	More than once a week	105 (4.64%)	21 (20%)	8 (7.6%)	4 (3.8%)	2.88 (1.70–4.70)***	3.04 (1.30–6.25)**	2.25 (0.66–5.82)	1.12 (0.53–2.29)	1.37 (0.44–4.02)	3.34 (0.56–16.5)
	Almost every day	94 (4.15%)	24 (25.5%)	8 (8.5%)	4 (4.3%)	3.95 (2.37–6.38)***	3.42 (1.46–7.08)**	2.53 (0.74–6.55)	1.12 (0.50–2.44)	1.50 (0.44–4.80)	2.23 (0.34–12.9)
(v) By who have you been cyber bullied?—Adult women	Never	2,009 (89.9%)	177 (8.8%)	61 (3%)	31 (1.5%)	Ref	Ref	Ref	Ref	Ref	Ref
	Less than once a week	97 (4.34%)	19 (19.6%)	11 (11.3%)	3 (3.1%)	2.52 (1.45–4.17)***	4.08 (1.98–7.75)***	2.04 (0.48–5.83)	1.18 (0.57–2.32)	1.83 (0.67–4.63)	2.14 (0.27–10.8)
	More than once a week	68 (3.04%)	16 (23.5%)	12 (17.6%)	2 (2.9%)	3.18 (1.73–5.57)***	6.84 (3.35–13.0)***	1.93 (0.31–6.57)	1.21 (0.54–2.62)	2.16 (0.76–5.85)	2.84 (0.32–16.7)
	Almost every day	60 (2.69%)	17 (28.3%)	2 (3.3%)	5 (8.3%)	4.09 (2.23–7.20)***	1.10 (0.18–3.64)	5.80 (1.92–14.3)***	1.42 (0.60–3.23)	0.13 (0.01–1.04)	8.43 (1.43–41.4)*
(vi) By who have you been cyber bullied?—Adult men	Never	2,005 (89.5%)	174 (8.7%)	57 (2.8%)	32 (1.6%)	Ref	Ref	Ref	Ref	Ref	Ref
	Less than once a week	113 (5.04%)	27 (23.9%)	11 (9.7%)	6 (5.3%)	3.30 (2.05–5.17)***	3.69 (1.79–6.98)***	3.46 (1.28–7.89)**	1.71 (0.90–3.18)	0.99 (0.32–2.72)	2.61 (0.53–11.3)
	More than once a week	52 (2.32%)	11 (21.2%)	10 (19.2%)	2 (3.8%)	2.82 (1.36–5.40)**	8.14 (3.70–16.4)***	2.47 (0.39–8.45)	1.49 (0.60–3.50)	5.63 (1.84–16.3)**	0.00 (0.00–2,164,986,805,448)
	Almost every day	70 (3.12%)	15 (21.4%)	8 (11.4%)	3 (4.3%)	2.87 (1.54–5.06)***	4.41 (1.88–9.15)***	2.76 (0.65–7.96)	1.21 (0.52–2.68)	2.41 (0.80–6.83)	0.91 (0.12–5.11)
(vii) By who have you been cyber bullied?—Person unknown to you	Never	1,871 (82.6%)	154 (8.2%)	56 (3%)	31 (1.7%)	Ref	Ref	Ref	Ref	Ref	Ref
	Less than once a week	196 (8.66%)	28 (14.3%)	13 (6.6%)	4 (2%)	1.86 (1.18–2.82)**	2.30 (1.19–4.16)**	1.24 (0.36–3.17)	1.07 (0.60–1.85)	1.02 (0.43–2.26)	1.22 (0.25–4.42)
	More than once a week	93 (4.11%)	22 (23.7%)	9 (9.7%)	4 (4.3%)	3.45 (2.04–5.64)***	3.47 (1.56–6.93)***	2.67 (0.78–6.92)	1.47 (0.72–2.88)	0.78 (0.25–2.15)	1.10 (0.14–5.55)
	Almost every day	104 (4.59%)	23 (22.1%)	5 (4.8%)	5 (4.8%)	3.17 (1.90–5.10)***	1.64 (0.56–3.80)	3.00 (1.01–7.24)*	1.47 (0.76–2.73)	0.59 (0.18–1.63)	1.74 (0.35–6.88)
(viii) By who have you been cyber bullied?—A group (e.g., a group of friends, a class etc.)	Never	1,829 (83.1%)	149 (8.1%)	56 (3.1%)	31 (1.7%)	Ref	Ref	Ref	Ref	Ref	Ref
	Less than once a week	156 (7.09%)	28 (17.9%)	15 (9.6%)	3 (1.9%)	2.47 (1.56–3.79)***	3.37 (1.80–5.96)***	1.14 (0.27–3.23)	1.55 (0.85–2.76)	1.06 (0.40–2.58)	0.25 (0.01–1.62)
	More than once a week	96 (4.36%)	21 (21.9%)	6 (6.3%)	2 (2.1%)	3.16 (1.85–5.18)***	2.11 (0.80–4.66)	1.23 (0.20–4.16)	1.74 (0.85–3.44)	0.71 (0.22–2.02)	0.47 (0.02–3.19)
	Almost every day	120 (5.45%)	28 (23.3%)	10 (8.3%)	8 (6.7%)	3.43 (2.14–5.35)***	2.88 (1.35–5.56)**	4.14 (1.74–8.81)***	1.71 (0.87–3.29)	1.30 (0.46–3.47)	1.38 (0.31–5.27)

*COR* Crude Odds Ratio, *AOR* Adjusted Odds Ratio, *CI* Confidence Interval, *Ref*. Reference category

^*^p < 0.05

^**^p < 0.01

^***^p < 0.001

^†^ = row percentage

#### Other associations with bullying

Being bullied outside school and being a girl victim of bullying were associated with suicide attempts without prior thoughts or plans. Being bullied by adults and group bullying were associated with thoughts proceeding to attempt without plans. Group bullying was associated with suicide attempts with prior thoughts and plans.

Suicidality in cyberbullying was associated with being bullied every day (for thoughts, plans and attempts), and being bullied by boys and by adult men (thoughts and attempts without plans). Cyberbullying by women was associated with suicidal attempts without thoughts and plans.

## Discussion

### Preamble

While previous studies have touched upon aspects of bullying, what distinguishes this research is its focus on examining the co-occurrence of face-to-face bullying and cyberbullying within the same cohort of Kenyan youth. The focus is on the perpetrator i.e. the bully in a way that can suggest the focus of the intervention. Furthermore, this study delves into how these forms of bullying are associated with various aspects of suicidality and socio-demographic characteristics. Therefore, being the first study of its kind reported in Kenya and for the purpose of sharing data for future research, this study endeavors to provide as much baseline data as possible.

This study presents the first Kenyan study with a wide spectrum of data on the comparative prevalence of different types of bullying (face-to-face and cyberbullying) in different settings, that is in school and outside school in the same cohort and at the same time. It confirms earlier findings from previous research referenced in the introduction that about 80% of high school students experience bullying and that bullying within schools is perpetrated by a smaller number of bullies [7, 8]. It also reports using a large sample that cyberbullying is prevalent in Kenya though less than face-to-face bullying. There is also a finding that bullying is associated with suicidality – that is, thoughts, plans and attempts but in different combinations and that not all who attempt suicide have suicidal thoughts and plans, suggesting attempt at the spur of the moment. This study establishes for the first time in the Kenyan setting that most of the significant associations and predictors of attempts are: outside school circles; by adults and specifically adult women; located at the family level in the name of siblings. Group bullying (inside or outside school) and the female gender are associated with suicidality in bullying. The study discusses all key findings and their practice and policy implications, within the Kenya context.

### Internal consistency

The tool used in this study was reliable. In addition, this tool has been extensively used as referenced in the methodology. Cronbach's alpha coefficient for the Suicidality subscale (which had 3 items) was 0.523. An Alpha value > 0.5 is considered acceptable for items less than 10 [32]. Thus, the 3 items within this subscale are correlated and measure the construct of suicidality.

For more than 10 items, the acceptable cut-off point is ≥ 0.65 [32, 33]. For the Bullying Experiences subscale (11 items), Cronbach's alpha was 0.708, suggesting acceptable internal consistency for this subscale. The Cronbach's alpha for Cyber Bullying Experience (8 items) was 0.862 (ranked as very good) [33].

### Social demographics

The finding that female gender is associated with suicidality in bullying agrees with previous literature as reported in the introduction [13]. These gender disparities in suicidality underscore the importance of gender-sensitive approaches in suicide prevention efforts. The gender disparity of 66.6% male and 33.2% female is a reflection of the schools recruited –more boy schools. The decreasing number with years in high school could be a reflection of dropout over time or due to the lack of availability of older students to participate in the study due to preparation for the national examination which was about to take place. The 61% rural as opposed to 38.7% urban is a reflection of the deliberated effort to reach rural schools. The higher prevalence of suicidality among certain age groups or rural populations could be attributed to unique social and environmental stressors prevalent in these contexts. While this study contributes valuable information on demographic correlates of suicidality, further research is needed to explore the underlying mechanisms driving these associations and to develop targeted interventions that address the specific needs of at-risk populations.

### Prevalence of bullying

Bullying in Kenyan high schools has remained consistent at around 80% as reported in the introduction suggesting that any interventions (the most significant intervention that has been put in place is “banning of bullying”) so far used have not been effective [7, 8]. This prevalence is similar to that of a study in Nigeria as reported in the introduction [6]. This calls for a multifaceted concerted effort to address bullying in school, community and home environments. An earlier study had proposed the following approaches: having bullying multifaceted interventions to address psycho-socio-behavioral problems; characterizing bullies and victims in terms of personality and environmental factors that may be associated with or conducive to bullying as well as determining the long-term prognosis for both bullies and victims [7]. This requires focused efforts on new students who are enrolled in high schools and new students from other environments where bullying is practiced.

It is not surprising that cyberbullying is less than face-to-face bullying a reflection of the limited access to the internet. However, this is set to increase given the increased availability of internet and electronic devices which were necessary for online classes during COVID-19, and have continued to be available and also attractive to youth following the pandemic [35].

### Association of bullying with suicidality

Past reported studies in Kenya [24, 34]have identified associations and predictors of suicidality. However, these studies did not consider bullying as one of the associated factors. The inclusion of suicidality in a study on bullying adds to factors associated with suicidality. This could well be a key consideration, given the high level of suicidality and the high levels of bullying that have been consistently found in Kenyan students [7, 8, 20].

Bullying is a traumatic event that leads to anxiety and depression as the conduits to suicidality as has been observed elsewhere [36]. This study has established multiple associations between bullying and suicidality, including social demographic predictors and associations inside and outside school which provide entry points for the intervention, especially those based at family and community levels.

### The different stages of suicidality

Whereas the most common progression of suicidality is from thoughts, plans and eventually attempts, this pattern is not always followed (37). Of concern is when attempts are made without prior thoughts and/or plans. Thus, it is important for teachers, parents, clinicians and even peers not to rely solely on suicidal thoughts or plans for intervention, but on other indicators such as the presence of bullying of different types, by whom and the environment which may precipitate attempt at the spur of the moment.

### A public health issue

The findings of this study suggest that bullying outside school rather than in schools is more likely to be associated with suicidality. Being bullied by an adult and in particular by an adult woman located either in school or outside school rather than a schoolmate, are significantly associated with suicidality. This could be a reflection of power imbalance, with the children feeling totally outweighed by adults. This is most likely to do with sexual harassment including incest and rape by adults, both men and women, within and outside the family circle, which was reported widely in social media during the COVID-19 lockdown. The finding of female gender as a predictor of suicidality could be explained in that the female gender is more vulnerable to sexual abuse, within and outside the family. However, it is essential to acknowledge the limitations associated with demographic variables, such as cultural differences in reporting, which may impact results interpretation. It is not surprising that bullying by sibling was a predictor of suicide. This could be as a result of sibling rivalry and in particular step siblings. Step siblings may perceive, rightly or wrongly that parents and guardians treat them differently. If one sibling – whether same or different biological parenthood performs better than the other in school, the lesser performing may take this negatively and feel the better performing sibling is, rightly or wrongly, bullying him or her. The better performing sibling may indeed tease the lesser performing. Alternatively, the less performing sibling may, out of jealousy, be hostile to the better performing sibling. This is more so if they happen to be in the same class, with rivalry being extended from school to home. Combined mixed methods may establish the actual dynamics. These findings therefore point to family circles, as fertile grounds for understanding and managing bullying in high school students. This is a responsibility that cannot be left to the teachers and/or clinicians alone. There is the need for public awareness of this aspect of bullying. Community based dialogue to address it at the community level has the potential to percolate down to the family level as well as reaching out to school environments without any family feeling that they are targeted.

## Conclusion

Based on the findings of the study, it is evident that bullying remains highly prevalent in students, with approximately 80% of students reporting various forms of bullying. Family and community-based bullying has a higher risk for suicidality compared to school-based bullying particularly when bullying occurs outside of school settings or involves adults, including instances of sexual harassment. As a result of this finding, taking a public health approach with the potential to primarily reduce bullying in the youth and secondly reduce suicidality in the same youth is recommended. The need for interventions at the family and community levels, as well as heightened public awareness and dialogue to address bullying dynamics within familial and school environments is also recommended. Recognizing that suicidal behavior may not always follow the typical progression of thoughts, plans, and attempts, it is crucial for stakeholders to be vigilant and responsive to indicators of bullying as potential precursors to impulsive suicidal acts. This study of bullying adds to the increasing number of predictors of suicide particularly those that can be manipulated for purposes of intervention. Furthermore, the study contributes to the global understanding and variability of suicidality in various contexts and is therefore a significant contribution to global mental health. All the aims summarized at the end of the introduction have been achieved.

## Limitations

This study was purely quantitative and was not based on clinical cases where there is one on one face-to-face evaluation. Furthermore, the students did not respond to any specific questions on cyberbullying based on our definition of cyberbullying but rather based on what the students understood and perceived to be cyberbullying and who the perpetrator was. Further mixed methods study designs to address issues such as definitions of bullying, common understanding of bullying, and whether these are influenced by the culture and levels of literacy, social desirability bias, and technological literacy are recommended. This would probably require several studies. A further limitation is that the tool used was an adopted instrument that had not been validated first and no psychometric properties provided for the adopted version. However, the tool has been extensively used in published literature. In mitigation, we used Cronbach's alpha to determine the reliability of the tool in this particular study.

## Data Availability

Requests for the data may be sent to the corresponding author.
